# Measuring Perceived Psychological Stress in Urban Built Environments Using Google Street View and Deep Learning

**DOI:** 10.3389/fpubh.2022.891736

**Published:** 2022-05-11

**Authors:** Xin Han, Lei Wang, Seong Hyeok Seo, Jie He, Taeyeol Jung

**Affiliations:** ^1^Department of Landscape Architecture, Kyungpook National University, Daegu, South Korea; ^2^School of Architecture, Tianjin University, Tianjin, China; ^3^School of Architecture, Harbin Institute of Technology (Shenzhen), Shenzhen, China

**Keywords:** deep learning, Google Street View, semantic segmentation, perceived psychological stress, built environment

## Abstract

An urban built environment is an important part of the daily lives of urban residents. Correspondingly, a poor design can lead to psychological stress, which can be harmful to their psychological and physical well-being. The relationship between the urban built environment and the perceived psychological stress of residents is a significant in many disciplines. Further research is needed to determine the stress level experienced by residents in the built environment on a large scale and identify the relationship between the visual components of the built environment and perceived psychological stress. Recent developments in big data and deep learning technology mean that the technical support required to measure the perceived psychological stress of residents has now become available. In this context, this study explored a method for a rapid and large-scale determination of the perceived psychological stress among urban residents through a deep learning approach. An empirical study was conducted in Gangnam District, Seoul, South Korea, and the SegNet deep learning algorithm was used to segment and classify the visual elements of street views. In addition, a human–machine adversarial model using random forest as a framework was employed to score the perception of the perceived psychological stress in the built environment. Consequently, we found a strong spatial autocorrelation in the perceived psychological stress in space, with more low-low clusters in the urban traffic arteries and riverine areas in Gangnam district and more high-high clusters in the commercial and residential areas. We also analyzed the street view images for three types of stress perception (i.e., low, medium and high) and obtained the percentage of each street view element combination under different stresses. Using multiple linear regression, we found that walls and buildings cause psychological stress, whereas sky, trees and roads relieve it. Our analytical study integrates street view big data with deep learning and proposes an innovative method for measuring the perceived psychological stress of residents in the built environment. The research methodology and results can be a reference for urban planning and design from a human centered perspective.

## Introduction

In contrast to the natural ecological environment, a built environment is a man-made environment characterized by land use, urban planning, and urban transport planning, including the building intensity and density, land use mix, street scale and articulation, and aesthetic quality of the urban landscape (1). In 2003, the *American Journal of Public Health* published a special issue on the “Built Environment and Health” theme (2). In the same year, the *American Journal of Health Promotion* also published a special edition on “Health Promoting Community Design” (3). This focus on health and the built environment by two leading journals in the health research field clearly indicates that the urban built environment is highly relevant to human health. Behavioral and temporal geography suggest the existence of a complex spatial relationship between daily human activity and the built environment. They imply that the perceived psychological stress and behavior of residents are conditioned and influenced by external spatial conditions (4, 5). Persistent stress can lead to anxiety, insomnia, and in some cases, psychological and physical illnesses. Stress-induced mental illnesses can be difficult to detect at first; however, if left unchecked, they can eventually have a significant impact on the body (6). A high-quality urban built environment helps promote outdoor activities, which lead to improved physical fitness, greater mental invigoration, psychological stress reduction, and fewer negative emotions and effectively reduce the incidence of many chronic diseases (7). The fact that a high-quality urban built environment reduces psychological stress and generates many other positive consequences has led academics and urban planners to focus more on the creation of high-quality people-centered built environments (8). However, this effort requires the measurement of the perceived psychological stress within a built environment; therefore, a method for quickly and effectively measuring this perception on a large-scale must be explored.

Several researchers from various fields have attempted to measure the perceived psychological stress of urban residents in a built environment (9–11). Using questionnaires to measure psychological stress is the most widely used approach because a traditional questionnaire design is based on various psychological theories (12). However, human psychological stress can also be monitored using specialized instruments. The physical appearance of an individual (e.g., facial expression and eye and head movements) can be employed as an indicator of his/her stress level, and individual perceived stress levels can be recorded with a high degree of confidence through human response monitoring and measurement (13). These methods can measure the perceived psychological stress in a built environment, but are not suitable for daily psychological stress monitoring because they are time consuming, costly, and inefficient. They also have a small sample size, and hence, are only suitable for small-scale studies rather than large-scale built environments.

The emergence of crowdsourced mapping services and geotagged imagery containing a wealth of visual information [e.g., Baidu Street View, Tencent Street View, and Google Street View (GSV)] provides a usable source of big data. Mapping services provide academics and researchers with an application programming interface (API) for extracting high-spatial resolution images of streets and communities to reflect a built environment as its residents see it (14). GSV is now available in many countries and cities around the world, becoming a scholarly tool for studying the built environment of cities because of its wide coverage and high accuracy. Moreover, with the rapid development of the computer technology, the use of deep learning is becoming increasingly widespread. Accordingly, a greater number of researchers are using deep learning to study the urban built environment and understand the street quality (15, 16).

In this work, the study area is Gangnam District in Seoul, South Korea. Google Maps has a better coverage of Korea than domestic companies like Kakao and Naver Maps; thus, it can be used to study Gangnam in more detail. We use GSV as the data source to determine the perceived stress levels in an urban built environment and achieve two main objectives. First, we employ deep learning to construct a SegNet architecture with a fully convolutional neural network to allow the pixelated semantic segmentation of street images from Gangnam and categorize the semantic segmentation results into visual elements used as explanatory variables for the perceived psychological stress in the built environment. Second, a random forest-based human–machine adversarial model is used to quickly score the perceived psychological stress of residents in the built environment within Gangnam on a large-scale, and stress distribution is investigated using an autocorrelation analysis. Three levels of perceived psychological stress (i.e., low, medium, and high) are identified in the GSV images. The relative abundance of specific visual constituents is measured to investigate the perceived stress characteristics in each GSV image. The relationship between the visual constituents of the urban built environment and the perceived psychological stress is assessed using multiple linear regression analysis. The positive and negative elements affecting the perceived psychological stress are identified. This analysis of street-view big data using deep learning can provide researchers and urban planners with more targeted data related to urban street perception, encouraging higher-quality urban planning.

## Related Work

### Traditional Methods for Assessing the Perceived Psychological Stress in an Urban Built Environment

Existing measures for perceived psychological stress tend to monitor changes in psychological perceptions and physiological signals in response to different stressors (17, 18). In an earlier study, experiments were conducted with a small number of subjects on the sensations of “oppression” and “release” experienced when moving through external urban spaces, and the results were examined and discussed by comparing changes in the environmental visual information of the surrounding scenes along the way (19). In traditional psychological research, psychological stress is measured using self-report questionnaires or by administering psychological scales. Peacock (20) used single-item, multi-question scales to measure the perceived psychological stress of a population in relation to specific stressors. A few scholars also scored the perceived psychological stress using expert interviews (21). These subjective measures are simple and easy to use, but susceptible to bias due to external interference and only somewhat reflective of an individual's psychological stress. When people experience external stress, changes in the brain signals and the nervous system lead to the release of hormones affecting physiological responses (e.g., heart rate, breathing, and blood pressure). In other words, the measurement of brain signals, stress hormones, and cardiovascular changes can provide an objective and real-time reflection of psychological stress (22). The development of science and technology has made it possible to employ brain wave detection, eye tracking, magnetic resonance imaging, and electrophysiological assessment to measure the perceived psychological stress in individuals, and these techniques have received extensive attention in psychological and sociological research. Aspinall et al. (23) conducted a series of experiments using mobile brainwave analysis to record and analyzes changes in the perceived psychological stress of urban residents in an urban street environment. Kacha et al. (24) used the Epoc Emotiv neuroheadset to conduct an exploration of electrobiological psychology based on the perceived complexity of street environments. Physiological signal detection is also widely used to measure psychological stress and avoid the limitations of the previous methods (e.g., questionnaires), indicating that the systemic changes in human psychological stress can be objectively represented (25). Although both physiological signal detection and traditional questionnaires can be used to measure the perceived psychological stress, they can be inefficient and time consuming, making them less useful for urban built environments over wide areas.

### Assessment of the Perceived Stress Based on Street-View Images and Deep Learning

Many international mapping services have made street-view data accessible to users through an API. The most popular mainstream mapping services used in research are Google Maps, Tencent Maps, and Baidu Maps (26–28). As interactive electronic maps, street views provide users with a panoramic view of the urban built environment at a low cost with high accessibility, high resolution, and wide coverage; thus, street-view images have become a very important new data source for urban built environment research (29–31). Salesses et al. (32) used thousands of geotagged urban street-view images to explore the perceived safety, class, and uniqueness of the built environment in the streets of Boston and New York in the United States and Linz and Salzburg in Austria. Li et al. (33) proved the potential of GSV imagery in depicting the built environment of cities to obtain a psychological perception consistent with that of city residents. The use of street-view images helps better explain the urban built environment characteristics and increases the level of understanding of this environment.

In recent years, many researchers have begun to use street-view images combined with deep learning algorithms to investigate the elements that most strongly affect urban street perception (30, 31). Deep learning algorithms, such as FCN, Resnet, and SegNet, use a deep convolutional neural network to process visual information within images, leading to an accurate identification of various visual features that include lanes, buildings, the sky, sidewalks, trees, and greenery and laying a solid foundation for better research on the quality of urban streets and human perception (34–36). Ordonez and Berg (37) collected a street-view image dataset from four cities and used deep learning models to explore a joint model of wealth, uniqueness, and perceived safety on a city scale. Meanwhile, in the Place Pulse project run by the MIT Media Lab, data from the collected street images were compared by visitors to an online website (http://pulse.media.mit.edu) on the evaluation dimensions of safety, liveliness, and wealth. This resulted in a deep learning dataset that is applicable to street measurements within a built environment (38). Tian et al. (39) extracted 30 street features from GSV and used deep learning algorithms to evaluate eight perceptual qualities, including ecology, closure, and accessibility.

Previous studies used big data from street-view images extracted using deep learning techniques to effectively measure the quality of an urban built environment. Attempts to link the visual elements of urban streets with the perceived stress of the residents have also been made. However, a complete research process for systematically analyzing the relationship between an urban built environment and the perceived psychological stress of residents or determining the autocorrelation of the perceived stress in space and the intrinsic mechanisms influencing this has not yet been developed.

## Methodology

An effective methodology for data collection, processing, and analysis was developed herein to meet the research objective of analyzing the relationship between an urban built environment and the perceived stress of residents. A systematic approach to the measurement of the perceived stress in urban environments was established using various techniques, such as automated street-view data collection and deep learning-based image semantic segmentation, which allowed the collection of accurate urban streetscape data and the analysis of perceived stress.

### Research Framework

**Figure 1:**
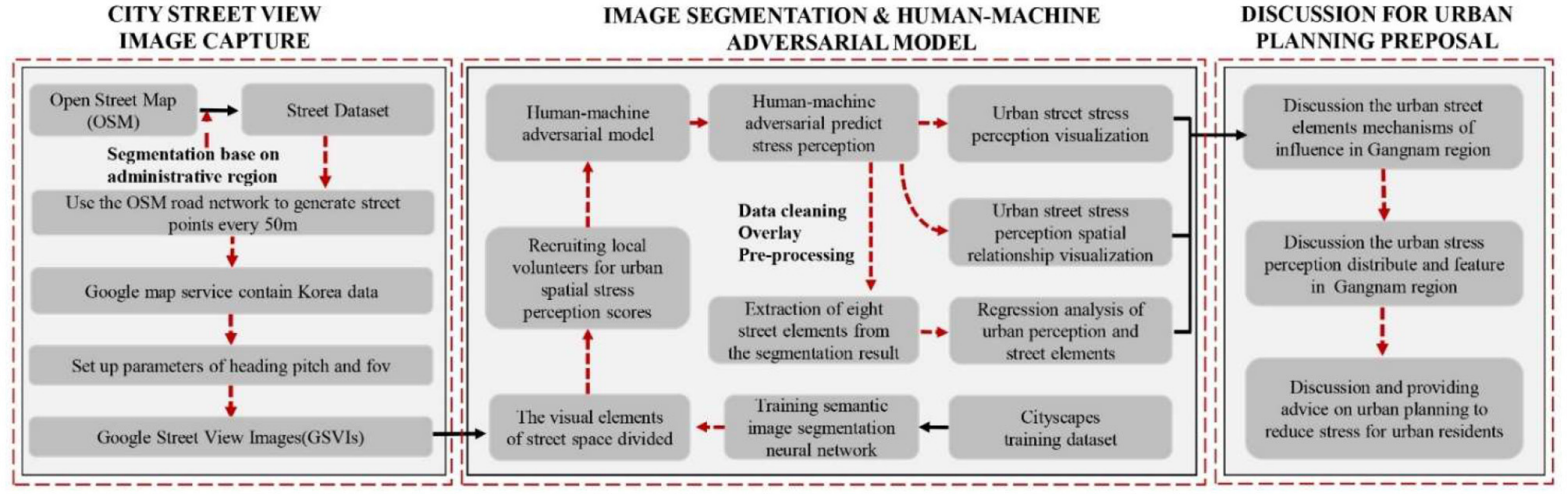
Analytical framework for the measurement of perceived stress in an urban built environment.

The research methodology consisted of three major stages (Figure 1). In the first stage, precise data for the street network of Gangnam were collected using OpenStreetMap (OSM). In ArcGIS, the road network data were employed to generate streetscape collection points at 50 m intervals. A Python program was then created to use Google Maps API to collect GSV images. In the second stage, a neural network model for semantic image segmentation was constructed using the Python-based TensorFlow framework. Accordingly, a full segmentation of the visual elements in the GSV images was conducted. The GSV images were processed using a human–machine adversarial model. Twenty volunteers were recruited to score the perceived psychological stress for the selected street scenes. In this case, the human–machine adversarial model predicted the remaining images to score the perceived psychological stress. The scores from each volunteer were weighted and averaged as the final score for the perceived psychological stress. The perceived psychological stress was then visualized in a map. In the third stage, a spatial correlation analysis of the perceived psychological stress in the urban built environment was conducted. A multiple linear regression analysis of the spatial visual elements of the built environment of streets was also performed to determine the positive and negative visual elements associated with the perceived psychological stress.

### Study Area

**Figure 2:**
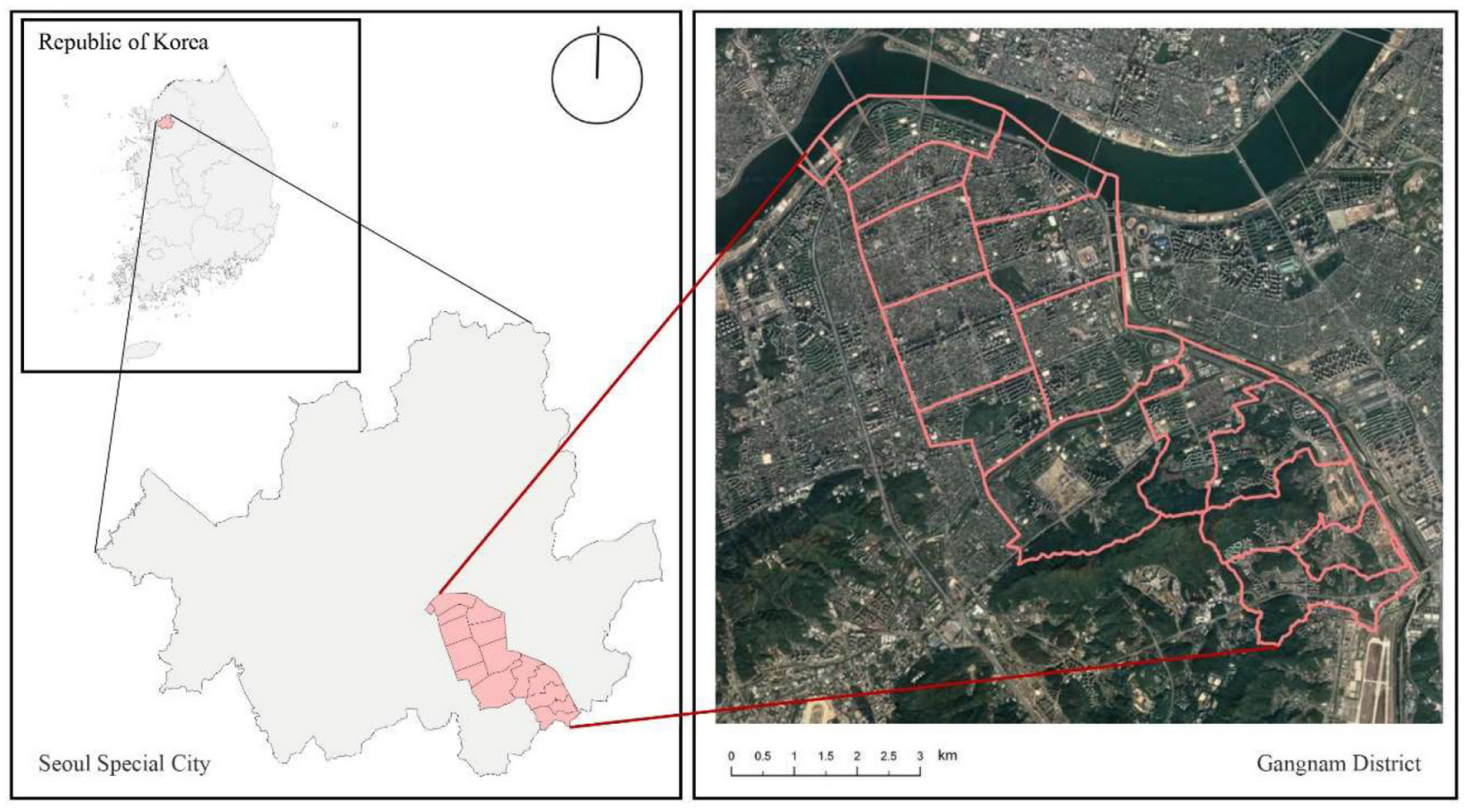
Study area: Gangnam District, Seoul, South Korea.

Our research was performed in Gangnam District, Seoul, South Korea (Figure 2). South Korea is located in the Korean Peninsula in East Asia, with Seoul as its capital city. Gangnam serves as an important commercial and residential area for the city's medium and upper classes. It is located south of the Han River and covers an area of 39.55 km^2^ with approximately 6,00,000 people. As one of the fastest-growing economic regions in Korea, Gangnam is ahead of other regions in Korea in terms of urban spatial regeneration. Due to its economic advantage, Gangnam has a representative urban built environment of a Korean metropolis. An empirical study of the Gangnam district can provide a preview of the perceived psychological stress problems that other Korean cities may face in their future development, and advices to other regions on the path toward urbanization to reduce residents' stress in urban construction. Therefore, Gangnam was selected as the case for this study on the perceived psychological stress of urban residents.

### GSV Image Collection

The use of streetscape images to analyze urban environments and various environmental psychological elements has become a common method in urban science research. These images allow for a quantitative measurement of a city from the perspective of an urban user. In addition to providing online street map services, most mapping service providers (e.g., Google) have released an API that allows the bulk customization of access parameters for street-view images.

**Figure 3:**
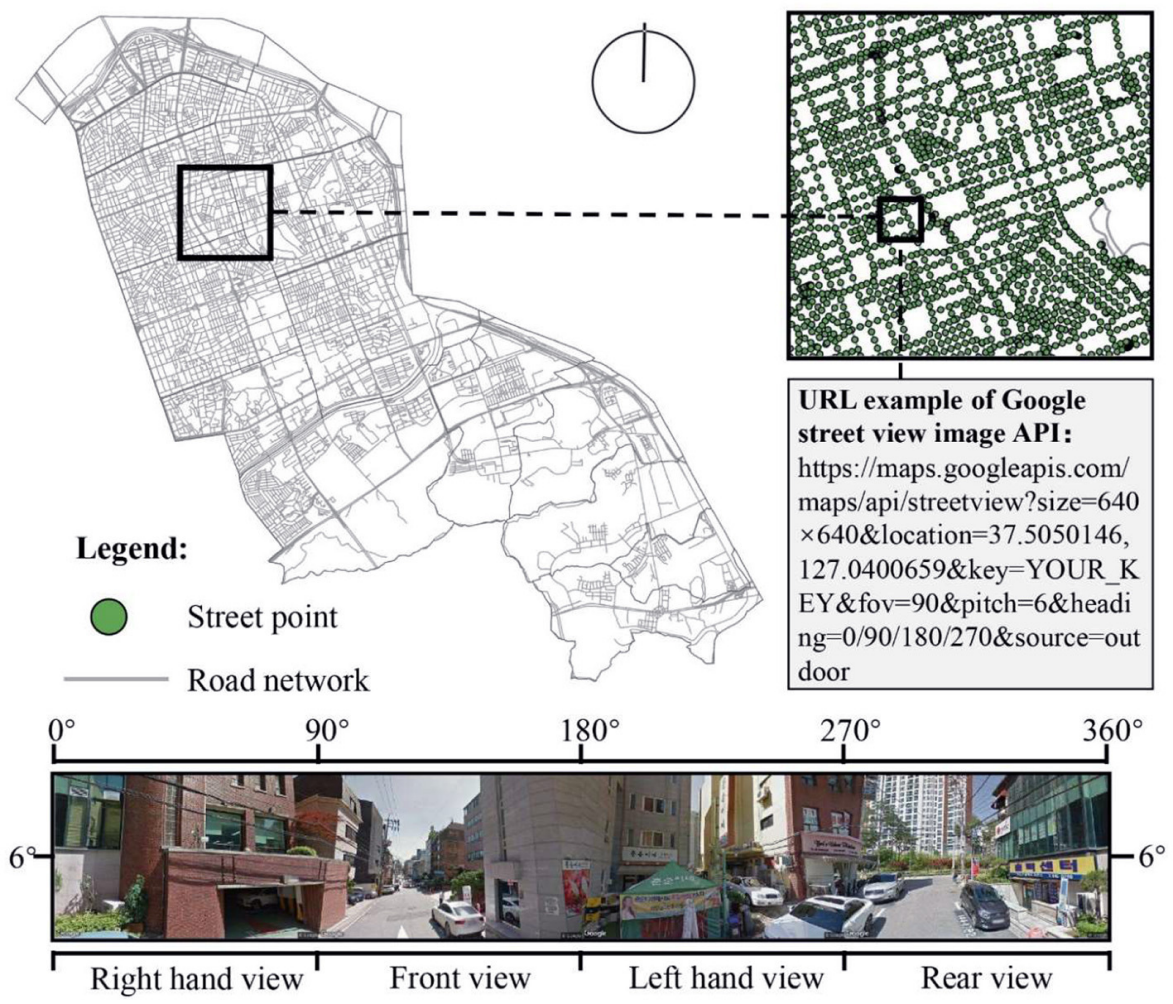
Example of a panoramic street-view image using Google Maps API.

For a complete recreation of the built environment in the streets of Gangnam District, panoramic GSV image collection points were set up and generated at 50 m intervals along the road network downloaded from the OSM. Consequently, 35,619 sample acquisition points were generated for the OSM street network in Gangnam. Figure 3 presents a portion of the Gangnam street network and an example sample point. The API was used to return the GSV images from the corresponding acquisition point as an HTTP URL. Static GSV images that matched the direction and angle of the human view within a limited range were obtained by adjusting the API and URL parameters for a standard HTTP request. In the GSV parameter settings, “location” represents the geographic latitude and longitude coordinates of the street-view capture point. We set the size of the obtainable image (Size) to a maximum of 640 × 640 px. The width of the field of view (Fov) representing a person was set to 90°. The street images were taken from four different angles (Heading). The field pitch angle (Pitch) was set to 6° to simulate the human perspective. Four GSV images (640 × 640 px each) were stitched together to provide a complete representation of the surrounding environment (bottom of Figure 3). The coordinates of the sampled points were input into a Python program that downloaded and stored 1,42,476 GSV images. The blank street-view images with invalid coordinate points were removed. This street-view data were used for image semantic segmentation and perceived stress calculations.

### Image Semantic Segmentation Based on Deep Learning

The Cityscapes dataset containing 34 types of objects from everyday life (e.g., sky, roads, autos, and plants) was chosen as the training dataset. Cityscapes contains urban street scenes from 50 different German cities, including Zurich, Hamburg, and Aachen. It comprises 5,000 high-quality pixel level annotated images of urban driving scenes (2,975 for training, 500 for validation, and 1,525 for testing, with 19 classes turned on by default for training in the dataset used for the study).

This study employed a classical image semantic segmentation method based on the SegNet codec structure, which is an open-source image segmentation project developed and published in 2015 by a team at the University of Cambridge. This deep learning algorithm can classify the semantics of objects in an image (e.g., sky, roads, and buildings) to the pixel level (40). SegNet consists of two main elements: an encoder and a decoder. The encoder primarily compresses and extracts object information. The decoder condenses the extracted semantic information to the input image size. Each pixel can be classified as the color of its corresponding object information.

In the SegNet network structure, several deep learning processing techniques are used to extract the streetscape image features. The images are resized to 416 × 416 using *Reshape*. The RGB values of the input image are employed for a feature three-dimension. The image two-dimensional (2D) matrix is padded with zeros to indicate rows and columns using *ZeroPadding2D*, which allows for better control over the feature map size and efficient feature extraction of the convolution kernel. The convolutional kernel *Conv2D* is employed to extract features from input high-dimensional arrays. Each feature map for the convolutional kernel output represents a filter, and the number of filters determines the number and depth of features extracted by the kernel. *BatchNormalization* has been proposed in (41) to normalize feature data to speed up gradient descent solutions, improve the network training speed, and increase the generalization ability. *Activation* introduces an activation function that gives the neural network a non-linear learning capability and enhances its ability to learn feature representations. Meanwhile, *MaxPooling2D* only uses operations in the encoder to dimensionalize the image and reduce the computational load. *UpSampling2D* is employed with the decoder to insert new numerical elements between pixel points, restoring the image to its original size. *Reshape* remaps the image to the size it was when input into the encoder. *Softmax* presents the results of multiple classifications as probabilities, calculating a specific semantic classification probability for each pixel across 19 categories.

**Figure 4:**
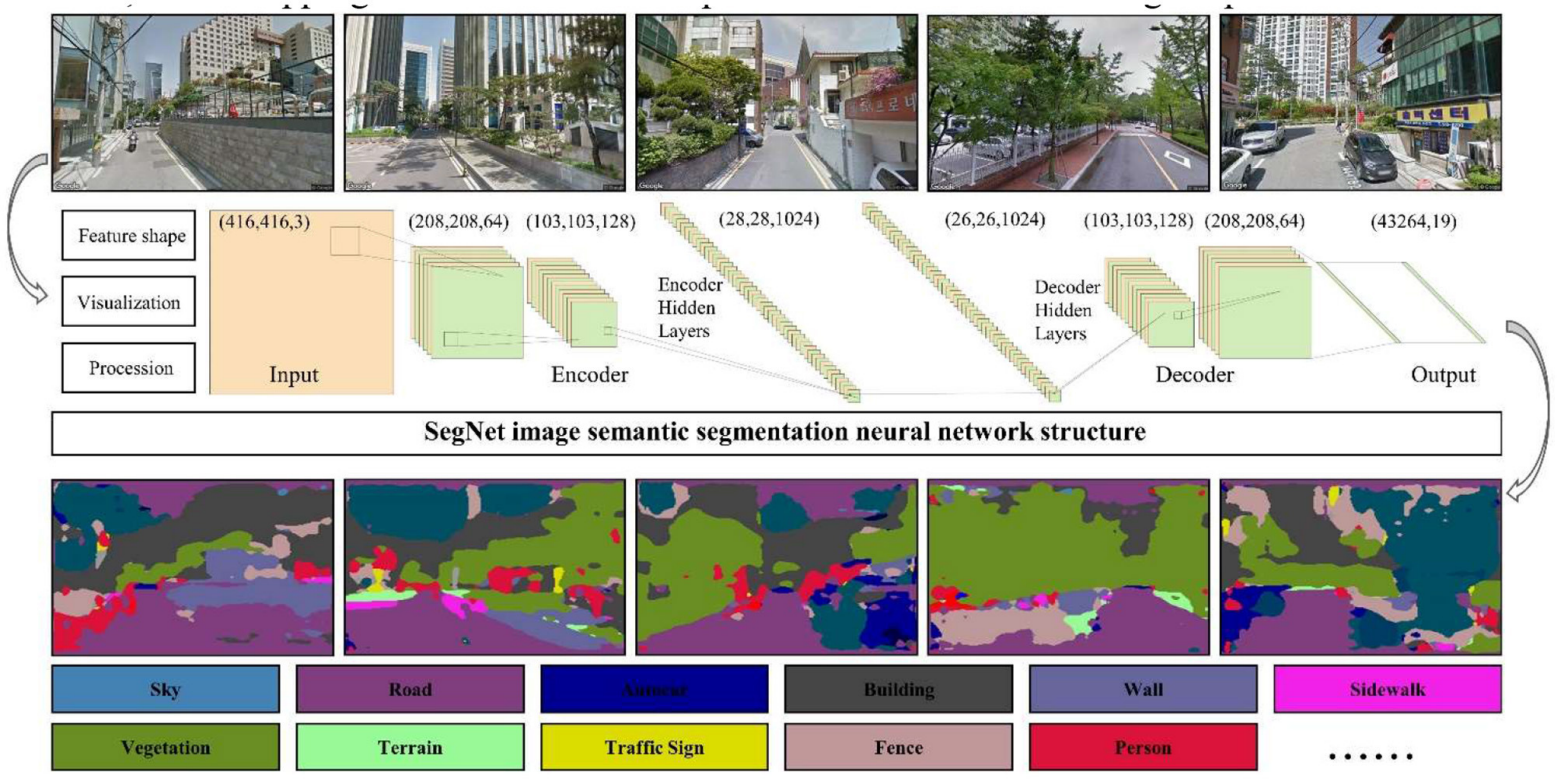
Visual elements extraction from urban street images using the SegNet image semantic segmentation model.

Figure 4 displays the original GSV images and the results of the segmented visual elements. The color matrix at the bottom represents the segmentation semantics corresponding to the extracted visual elements. The image semantic segmentation process using SegNet is also illustrated via a multi-level representation of the neural network structure, including the feature size of the codec neural network (“Feature shape”), visual representation of the neural network structure (“Visualization”), and image processing (“Procession”). The final image size and dimensionality are reduced, and a mapping classification of the specific semantics for the image is provided.

### Determining Perceived Psychological Stress Based on a Human–Machine Adversarial Model

We used the scoring framework for the human–machine adversarial model proposed by Yao et al. (42) to explore the perceived psychological stress of urban residents in a built environment. This deep learning framework utilizes a combination of iterative feedback and recommendation scores used to effectively assess the perceived psychological stress of the built environment in Gangnam. At the preliminary data preparation stage, we used Google Maps API to download the GSV images of Gangnam, processed the street-view images using the SegNet image semantic segmentation method, obtained the percentage coverage of the visual elements for each street-view image, and developed a 19-dimensional visual element feature vector for the human–machine adversarial model. To accommodate the scoring process, in which users scored parts of the street-view images, the human–machine adversarial model created a random forest (RF) dataset. In the RF model, a bootstrapping process randomly selects two-thirds of the sample for data fitting or classification. The remaining one-third is defined as out-of-bag (OOB) data used to reduce the overall model error and improve the variable importance. If *X*_*j*_ is used as an input variable, calculating the importance of *X*_*j*_ in the Nth tree *VI*_*n*_ will require the use of the sample data drawn from the bootstrapping process to create a regression tree model *T*_*n*_. This will be followed by the prediction error calculation for the OOB data, which eventually replaces the observations for variable *X*_*j*_ at random. Model $T_{n}^{\prime }$ is rebuilt, and the prediction error for the OOB′ data is calculated. The mean of all regression tree results represents the importance of variable *X*_*j*_ in the Nth random tree *VI*_*n*_(*X*_*j*_) after processing the prediction error of the two OOB datasets (43):


$$\begin{array}{l} VI_{n}\left(X_{j}\right)=\{\overset{N_{OOB\;}}{\underset{i=1}{\sum }}I\left[f\left(X_{i}\right)=f_{n}\left(X_{i}\right)\right]\\ \;-\overset{N_{OOB\;}}{\underset{i=1}{\sum }}I\left[f\left(X_{i}\right)=f_{n}\left(X_{i}^{\prime }\right)\right]\}/N_{OOB} \end{array}$$

**Figure 5:**
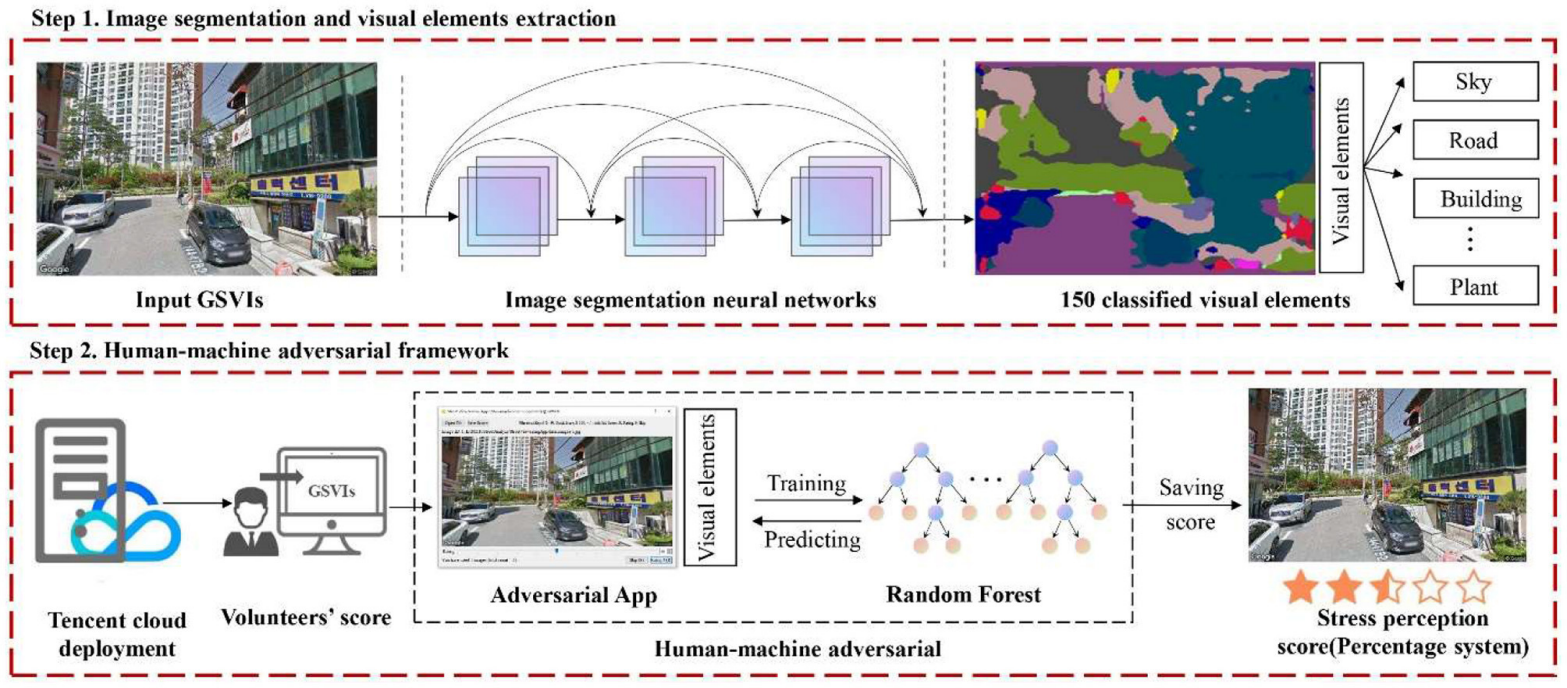
Perceived stress calculation for urban streets based on a human–machine adversarial model.

Humans have superior image recognition capabilities (44), that provides theoretical support for the use of human–machine adversarial models. Twenty Korean university students and staff were recruited as study volunteers (approximately 1:1 male : female ratio; age range: 19–52 years old) to measure the perceived psychological stress in the built environment in Gangnam. Volunteers accessed a human–machine confrontation model through the Tencent Cloud server to score the perceived psychological stress in the urban built environment. We require 15 s for each image to be displayed on the screen according to the standard of previous scholars for scoring time in image experiments (45, 46), during this period volunteers are able to perceive the content of the pictures deeply. We categorized the volunteers' perceived level of psychological stress when they saw the street view images, using numbers to quantify the criteria, scores from 0 to 100. Specifically, the street view produced a score of 100 for extremely high stress perception and a score of 0 for an extremely relaxed psychological state. These scores were used to measure the perception of urban stress. We used a non-complete random order for image extraction scoring to reduce the dataset error to some extent. Each volunteer rated the perceived psychological stress of 50 street-view images, and the results of which were recorded by the model. Subsquently, an RF dataset was created. Starting with the 51st street image, the model predicted the volunteer's perceived psychological stress score based on the relationship between the volunteer's previous scores and the corresponding street elements. If a difference is found between the score recommended by the model and the volunteer's subjective score, the model continues learning. If the error between the recommended score of six street-view images in a row and the subjective score of the volunteer exceeds 10, the model re-collects the volunteers' scoring features and continues the learning process until the error between the recommendation and subjective intention scores becomes < 10. The adversarial scoring between humans and a model represents a more stable algorithmic training approach. Figure 5 presents a schematic diagram of the evaluation process of the perceived psychological stress in an urban built environment using a human–machine adversarial model.

### Spatial Autocorrelation Analysis of the Perceived Psychological Stress in an Urban Built Environment

In the first law of geography, objects that are closer together are more strongly interconnected than those that are further away (47). Global spatial autocorrelation is defined as the characterization of a geographic attribute across a regional space and is used to measure the degree of association between spatial objects across a study area to determine whether there is a significant spatial distribution pattern exists for these objects. Statistics, such as global Moran's *I*, global Gear's *C*, and global Getis-Ord *G* are commonly used for this analysis (48). The present study employed global Moran's *I* to measure the global autocorrelation of the perceived psychological stress in the built environment in Gangnam using the following equation:


$$\begin{array}{l} I=n\overset{n}{\underset{i=1}{\sum }}\overset{m}{\underset{j=1}{\sum }}w_{ij}\left(x_{i}-\bar{x}\right)\left(x_{j}-\bar{x}\right)/\overset{n}{\underset{i=1}{\sum }}\overset{m}{\underset{j=1}{\sum }}w_{ij}{\left(x_{i}-\bar{x}\right)}^{2} \end{array}$$

Standardized *Z* values were used to test the significance level of global Moran's *I* based on the following equation:


$$\begin{array}{l} Z_{\;}=\frac{I-E\left(I\right)}{\sqrt{VAR\left(I\right)}} \end{array}$$

where *I* is the global Moran index; *n* is the total number of the study sites; $\bar{x}$ is the mean perceived psychological stress score; *x*_*i*_ and *x*_*j*_ are the perceived psychological stress score for regions *i* and *j*, respectively; and *w*_*ij*_ is the spatial weighting coefficient for regions *x*_*i*_ and *x*_*j*_, which reflects the relationship between regions *i* and *j* in space. If the regions are adjacent, *w*_*ij*_ = 1; otherwise, *w*_*ij*_ = 0. E(*I*) and VAR(*I*) denote the expected value and the variance of Moran's *I*, respectively. Global Moran's *I* has a range of [−1, 1]. A global Moran's *I* > 0 (p < 0.05) indicates a positive spatial correlation, that is, the high (or low) values for the perceived psychological stress are spatially significantly clustered. If it is equal to or close to 0, no spatial autocorrelation exists in the adjacent regions. In other words, psychological stress is randomly distributed. If it is lower than 0 (p < 0.05), a negative spatial correlation exists (i.e., the perceived psychological stress in adjacent areas is vastly different). At a significance level of 0.05, |Z|>1.96 means that global Moran's *I* is significant (49). Table 1 summarizes the relationship among the Z- and *p*-values and the significance level.

**Table 1:** Z- and *P*-values, and significance level.

**Z**	***P***	**Significance level**
Z < −1.65 or Z > 1.65	<0.10	90%
Z < −1.96 or Z > 1.96	<0.05	95%
Z < −2.58 or Z > 2.58	<0.01	99%

Global spatial autocorrelation can be used to describe the degree of autocorrelation for the perceived psychological stress in the built environment across the entire Gangnam District, but it cannot effectively express the spatial autocorrelation of different spatial units in Gangnam with adjacent areas. In 1995, Anselin (50) proposed the Local Indicators of Spatial Association (LISA) to examine the local spatial autocorrelation of individual spatial units. The LISA decomposes global Moran's *I* into spatial elements and forms a LISA aggregation map via Z-tests. The map reflects the specific locations where the spatial aggregation or divergence of variables within the study area occurs, thereby identifying areas with a strong influence on global associations as follows:


$$\begin{array}{l} I_{i}=\frac{\left(x_{i}-\bar{x}\right)}{S^{2}}\underset{j}{\sum }w_{ij}\left(x_{j}-\bar{x}\right) \end{array}$$

## Experiments and Results

In this section, we will first introduce the parameters of the platform on which deep learning techniques operate, such that other researchers can use it as a reference for their experiments. The deep learning training model records the number of training rounds and the dataset accuracy and validates the model's feasibility in image semantic segmentation by assessing various metrics. The index assessment also validates the RF scoring procedure, which is based on the human–machine adversarial model. The results reflect the perceived psychological stress of urban residents. The final perceived psychological stress scores in the urban built environment are spatially mapped.

### Image Semantic Segmentation Model Training Results

For the training and implementation of the deep learning and machine learning methods used in this study, the computer training variables were made consistent, and the training was conducted on the same Windows computer with an NVDIA GeForce GTX1070 graphics card, an AMD Ryzen5 2600X Six-Core Processor, 3.60 GHz, and 16 GB of RAM.

The Cityscapes dataset used to train the network has 2,975 and 500 images for training and validation, respectively. The batch size for each input was set to 2 to prevent memory overflow. Transfer learning was utilized in the training phase, in which the image feature extraction capabilities acquired while learning other tasks were transferred to help solve new problems (51). This method is widely used in the processing of remotely sensed (52), natural (53), and medical (54) images. Transfer learning significantly reduces training time, improves network model generalization, and prevents overfitting by sharing the learned image representation between models. The transfer learning model used herein had a weighting base of ResNet50 and was configured to first freeze the neural network at layer 142 and earlier (representing the transfer learning training phase), train for 20 rounds, and then unfreeze all network layers and continue with global training for 40 rounds, totaling to 60 training rounds.

The input image remapping window was set to 416 × 416 px for the neural network. The learning rate is reduced by half if no reduction in the loss happens after three rounds. The earl-stopping training method was used to stop network training when no reduction in the loss is observed over 10 rounds. In the transfer learning training phase, the Adam optimizer (55) was used with 0.001 learning rate. The same optimizer was used in the global learning training phase with 0.0001 learning rate. We employed the TensorBoard module in Keras to record the training data. Accordingly, 113 min was required for transfer learning training, and 281 min was required for the global learning training, accounting for a total of 6 h and 34 min of training completion. The code used for the experiment can be downloaded from GitHub (https://github.com/landscapewl/Segnet-Transfer-Learning).

**Figure 6:**
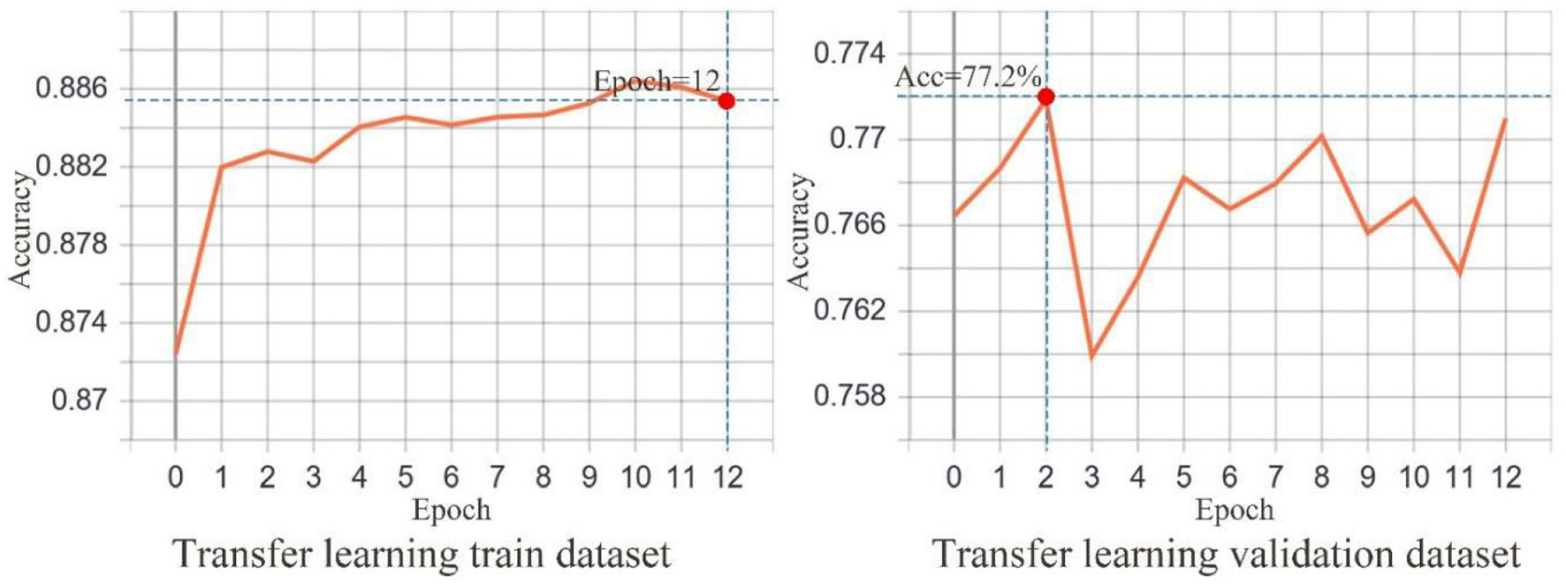
Transfer learning training accuracy. The number of epochs was set at 20. The graph on the left presents early stopping at epoch 12. The graph on the right shows a maximum accuracy of 77.2%.

**Figure 7:**
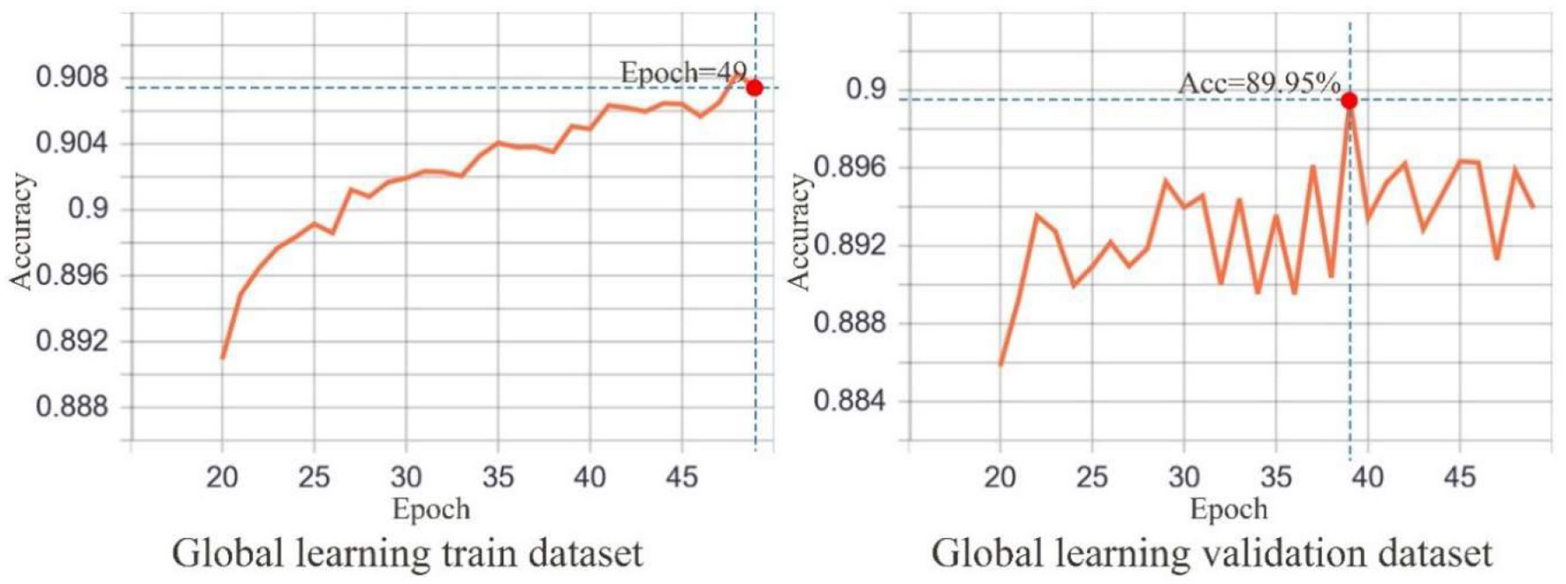
Global learning training accuracy. The number of epochs was set at 60. The graph on the left presents early stopping at epoch 49. The graph on the right shows a maximum accuracy of 89.95%.

The trained SegNet exhibited a better natural image scene segmentation performance. Using the Cityscapes dataset, our trained SegNet achieved 88.64% and 77.2% accuracies for the training and validation datasets, respectively, during the transfer learning phase (Figure 6). Figure 7 shows that the training dataset was segmented at 90.83% accuracy compared to 89.95% for the validation dataset during the global training. Figure 4 depicts the semantic segmentation results of the streetscape images from Gangnam using the trained network. The quality of the built environment in Gangnam greatly varied from the areas under construction to those with high-quality infrastructure; however, the model we trained demonstrated a satisfactory generalization in segmenting and resolving the complex arrangement of visual elements within the Gangnam street-view images.

### Accuracy Testing of the Human–Machine Adversarial Model

66.7% of the data scored by the volunteers was used to train the random forest model. The remaining 33.3% was used to detect the accuracy. The average error of the urban built environment psychological stress perception results was 1.33%. The RMSE was 2.86, the OOB error was 5.02%, and the OOB RMSE was 7.22. It can be seen that the accuracy of the random-forest-based estimation of the psychological stress perception for the urban built environment was over 95%, proving its excellent performance of the proposed model in predicting human perception.

### Perceived Psychological Stress in the Built Environment in Gangnam

After data cleaning, the invalid coordinate point crawl of the streetscape blank map was eliminated, leaving behind 31,378 coordinate points of the streetscape images. To more clearly show the stress perception level of Jiangnan District, the natural breakpoint method (56) was used to divide the psychological stress perception scores into six intervals (Table 2).

**Table 2:** Statistical summary of the perceived stress scores for 31,378 street-view image acquisition points from Gangnam.

**Level**	**Stress classification**	**Score range (%)**	**Number of acquisition points**	**Percentage of dataset**	
1	Low	10.00 to 35.00	6210	19.79%	34.82%
2		35.10 to 40.00	4717	15.03%	
3	Medium	40.10 to 45.00	6820	21.73%	33.96%
4		45.10 to 50.00	3840	12.23%	
5	High	50.10 to 55.00	4265	13.59%	31.18%
6		55.10 to 86.00	5520	17.59%	

**Figure 8:**
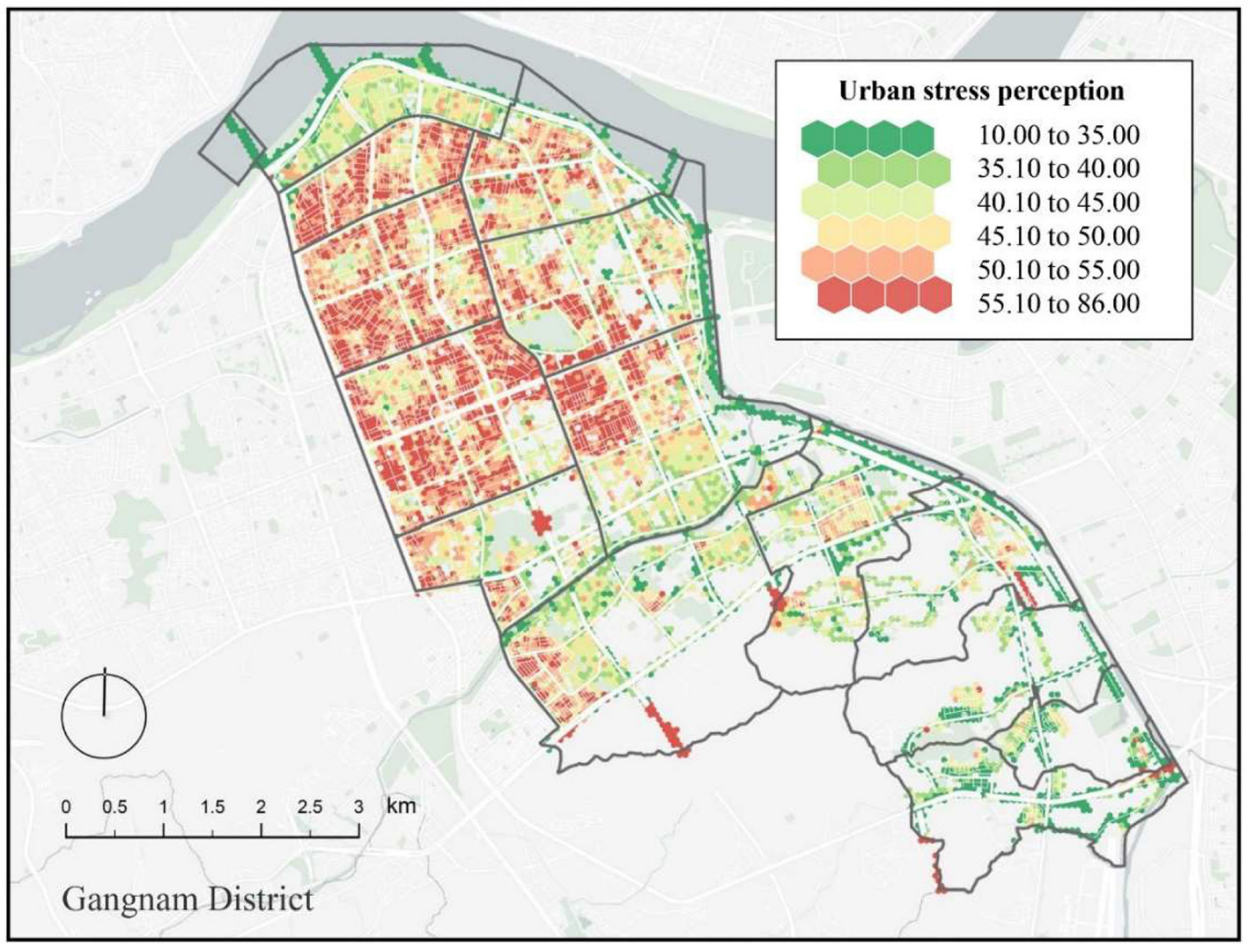
Urban spatial mapping of the perceived stress scores in Gangnam.

The Figure 8 legend depicts a gradual change from green to red showing the change in the pressure perception in Gangnam District. The stress perception distribution on this map significantly varied across different parts of Seoul. We connected the city stress perception score sampling points to a hexagonal grid because a hexagonal cell grid shares more adjacent edges than a quadrilateral, and the distances between the centers of mass of adjacent cells are equal. This allows the hexagonal grid to be more flexible in setting its parameters (e.g., radius) and for smoother transitions when representing the pressure-aware attribute distribution in urban space. The distribution of high stress perceptions is more mainly concentrated in the northwestern area of Jiangnan District. These areas are mostly residential and commercial sites. The small spacing between buildings and the lack of sufficient greenery planting in some streets may have contributed to the high perception of psychological stress among residents in this area. Medium to low stress perceptions are mainly found in the northern riverside areas and most of the southern areas. The northern riverside area is a high-class residential area and a green park with a higher degree of greenery and scenic beauty than the general residential area. This attribute may contribute to the low perception of stress among residents in this area. The southern region is more mountainous without many high-rise buildings and has a beautiful natural environment; therefore it does not generate too high a perception of psychological stress. To a certain extent, being close to nature helps reduce the perception of urban stress.

**Figure 9:**
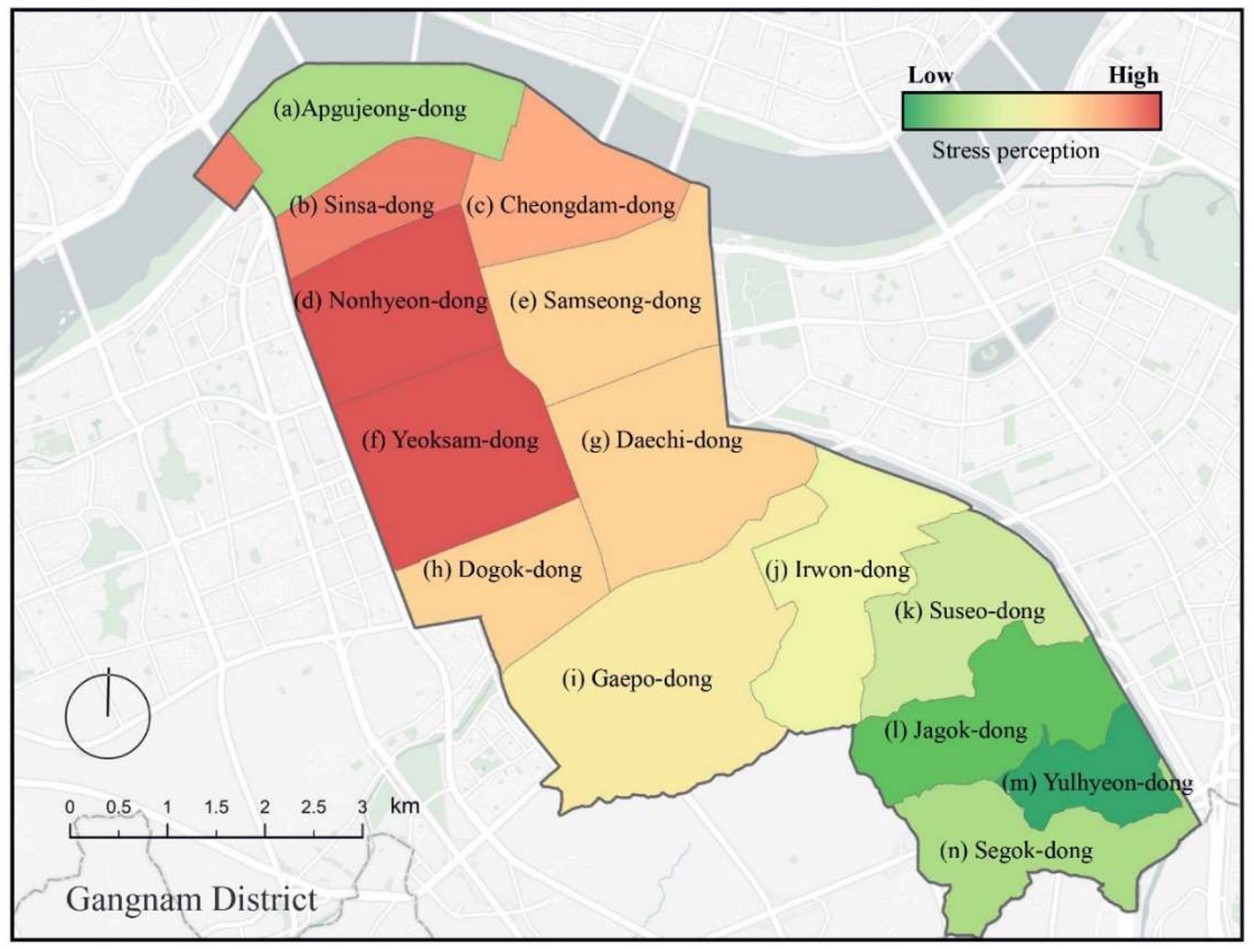
Urban spatial mapping of perceived stress for Gangnam administrative divisions.

Figure 9 illustrates the average urban perceived stress by administrative division within the Gangnam district. By studying the heterogeneity of the perceived stress for an urban built environment in this form, it is possible to distinguish administrative districts with high perceived stress from those with low perceived stress. Four administrative areas with high-perceived stress were identified: Sinsa-dong, Cheongdam-dong, Non-hyeon-dong, and Yeoksam-dong. Four administrative areas with medium-perceived stress were also determined: Samseong-dong, Daechi-dong, Dogok-dong, and Gaepo-dong. The remaining six administrative areas had low perceived stress: Apgujeong-dong, Irwon-dong, Suseo-dong, Jagok-dong, Yulhyeon-dong, and Segok-dong. The results thus show that the perceived level of stress in the central area of Gangnam was significantly higher than the surrounding areas. Similarly, the areas with low perceived stress were clustered in the southern region of Gangnam.

### Spatial Autocorrelation Analysis of the Perceived Psychological Stress

Most previous studies on perceived psychological stress in a built environment have focused on small areas due to the limitations of their research methodology. Knowledge of the distribution of perceived psychological stress in large-scale built environments is relevant for urban planning, urban regeneration, and urban management. Therefore, it is particularly important to visualize the spatial distribution of perceived psychological stress. Spatial autocorrelation models can measure the potential interdependence between point data at one location and other neighboring locations, enabling more effective statistical analysis of spatial data and the exploration of correlations between elements, which can help urban planners to take specific measures to address high perceived psychological stress in certain areas.

The global spatial autocorrelation analysis was performed here using the spatial autocorrelation function in ArcGIS. Table 3 summarizes the specific implementation methods. The FIXED_DISTANCE approach analyzed each element in the neighborhood. The proximity elements within the distance threshold were assigned a weight of 1 and affected the target element calculation. By contrast, the proximity elements outside the distance threshold were assigned a weight of 0 and did not affect the target element. The MANHATTAN distance method was used to measure the distance between two points along the vertical axis by summing the difference between the *x* and *y* coordinates. ROW normalization was conducted by dividing the weights by the sum of the weights of the neighboring elements, thereby reducing the potential for error. The minimum distance that ensured each element had at least one neighborhood was used as the threshold distance (85.7369 m).

**Table 3:** Summary of the data processing information for global Moran's *I*.

**Specification**	**Implementation**
Conceptualization of spatial relationships	Fixed_distance
Distance method	Manhattan
Standardization	Row
Distance threshold	85.7369 m

**Figure 10:**
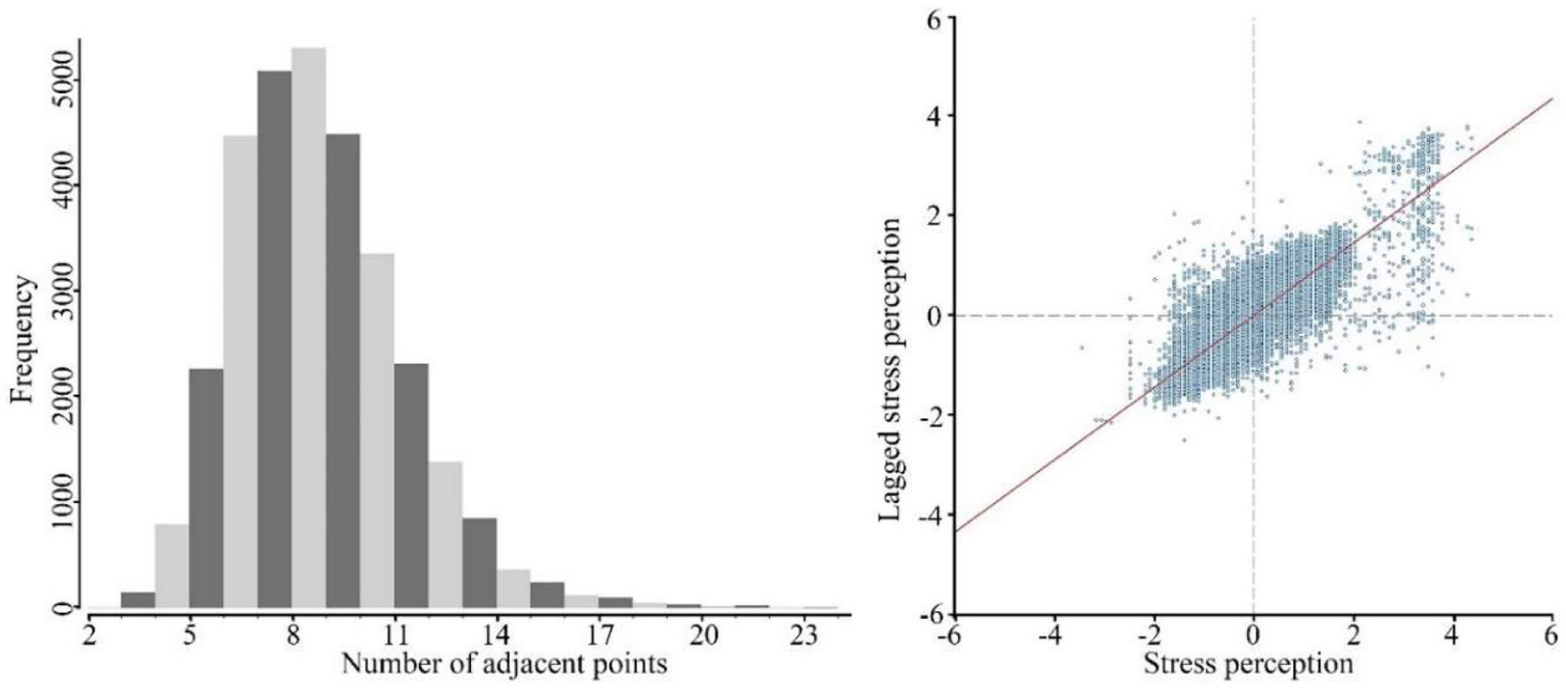
Left: bar chart of the number of perceived psychological stress neighbors. Right: Moran's*I*scatter plot.

Based on global Moran's *I* analysis, a Moran's *I* scatter plot and bar chart showing the number of perceived psychological stress neighbors was obtained (Figure 10). Most of the points in the scatter plot were distributed in quadrants 1 and 3, indicating that there was a positive spatial autocorrelation in the perceived psychological stress. The bar chart shows that the frequency of the number of neighbors was roughly in line with a normal distribution, indicating that the points are in space in line with the general statistics of the thing. In the global Moran' s *I* aggregate, the Moran' s *I* index was 0.655442. The Z value was 331.348650 (*p*-value < 0.01). There was 99% certainty that there was spatial autocorrelation in psychological stress perception and that psychological stress perception values were significantly clustered spatially. However, how the perceived psychological stress is clustered in space and what the distribution patterns are need to be addressed using local spatial autocorrelation.

**Figure 11:**
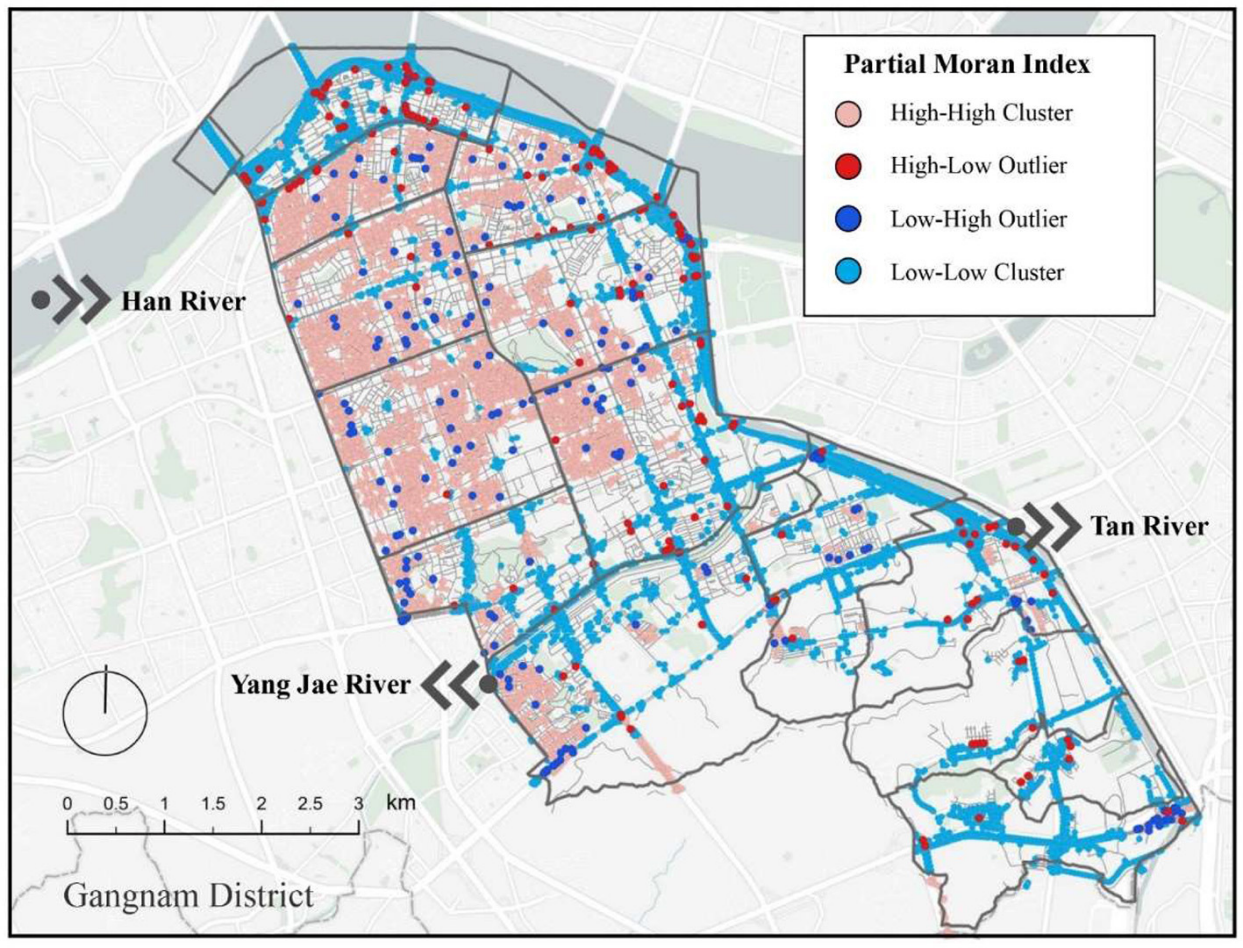
Spatially localized autocorrelation distribution map for Gangnam.

Figure 11 visualizes the results of the spatial local autocorrelation analysis of the perceived psychological stress in the built environment. The street acquisition points that were not spatially autocorrelated were excluded. The points with spatial aggregation relationships were classified into four categories of high–high, high–low, low–high, and low–low clusters. The high–high perceived stress clusters were clearly distributed in the northern area bounded by Yangjae River, which is mostly a residential area. This may be because the high land prices in Gangnam, which is one of the most developed areas in South Korea, make the residential areas more crowded. Moreover, the spaces between houses are narrow. The architectural style also does not reflect the local characteristics, and one can see the lack of a rational division of group architecture and spatial hierarchy, as well as of sufficient greenery to ensure the needs of the residents. The south shows low–low clusters along the main traffic routes, which may be due to the many mountainous areas with a beautiful natural scenery in the south, and the many parks in Gangnam that are located alongside the rivers and streams, where residents can go for walks, rest, and engage in other daily activities.

### Percentage Coverage of Visual Elements in Images From Low-, Medium-, and High-Stress Areas

Table 4 lists the eight visual elements from the image segmentation process that had the highest impact on the perception of psychological stress in the built environment.

**Table 4:** Statistics for the segmented results of the top eight GSVIs.

**Number**	**Visual element**	**Mean**	**Max**	**Min**	**S.D**.
1	Road	0.304	0.480	0.001	0.105
2	Sky	0.293	0.495	0.001	0.090
3	Building	0.150	0.560	0.001	0.108
4	Vegetation	0.068	0.496	0.001	0.073
5	Sidewalk	0.053	0.449	0.001	0.059
6	Auto	0.022	0.291	0.001	0.026
7	Wall	0.008	0.313	0.001	0.025
8	Grass	0.003	0.162	0.001	0.008

**Figure 12:**
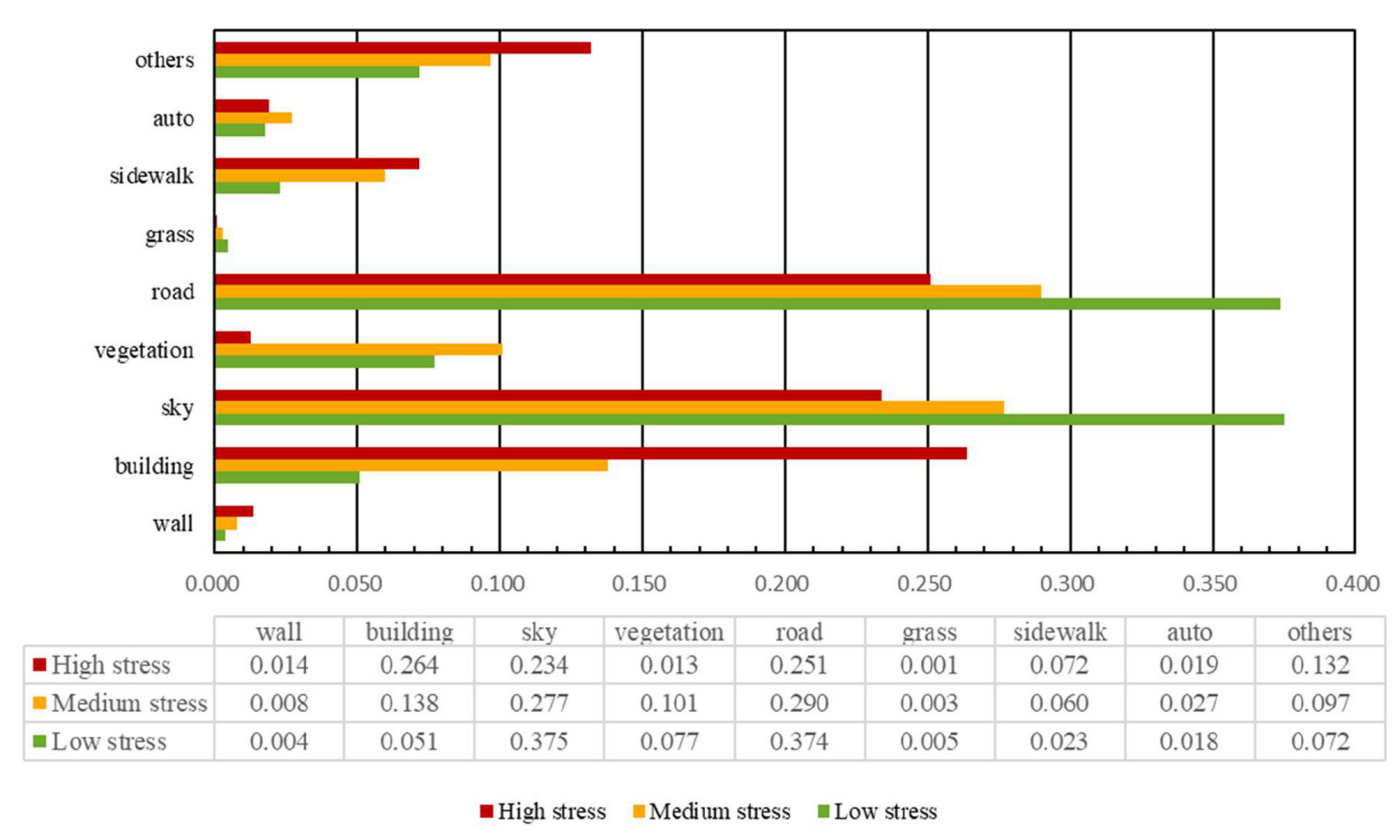
Proportional coverage of visual elements in images with low, medium, and high perceived stress.

In Figure 12, the perceived stress scores were divided into high, medium, and low to better understand the relationship between the proportional coverage of the visual elements and perceived stress. As the stress level increased, the average coverage of buildings in the images rose from 5.1 to 26.4%. An increase in building coverage constricts the street space, and the more enclosed space may oppress the residents. More buildings will inevitably crowd out the sky, roads, and vegetation, thus generating negative emotions. In addition, the percentage of both walls and sidewalks showed a small percentage increase from 0.4 to 1.4% and 2.3 to 7.2%, respectively. This is closely related to the laws of urban construction, with more buildings leading to more walls dividing the space and sidewalks connecting traffic. In contrast, as stress levels rose, there was a clear decrease in the percentage coverage of the sky and roads from 37.5 to 23.4% and from 37.4 to 25.1%, respectively. This means that an open sky and spacious roads cause human to feel more relaxed. Interestingly, the percentage coverage of vegetation increased from 7.7 to 10.1% when moving from low to medium stress and then decreased to 1.3% with high stress. This suggests that the relationship between perceived stress and vegetation is not linear and that there is a specific amount of vegetation that minimizes perceived stress. This is worth investigating in future research.

**Figure 13:**
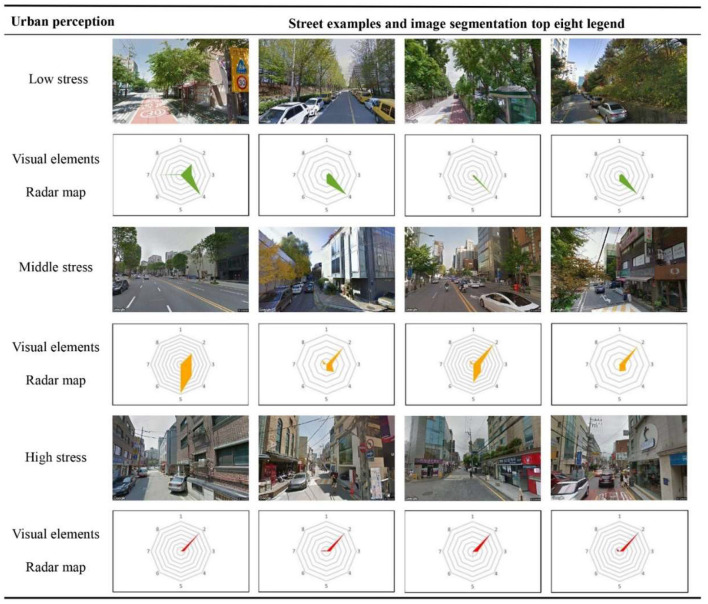
Representative images for different levels of perceived stress. The hexagon represents the eight image segmentation elements, clockwise from the vertex: (1) walls, (2) buildings, (3) the sky, (4) vegetation, (5) roads, (6) grass, (7) sidewalks, and (8) auto.

Figure 13 presents a selection of street-view images representing different perceived stress levels. The accompanying radar maps clearly show that the proportion of visual elements significant influences the stress levels. The proportion of auto, buildings, the sky, and roads significantly change between areas with low and high stress levels. In contrast, the percentage coverage of grass, sidewalks, and auto was relatively stable.

### Multiple Linear Regression Analysis of the Perceived Psychological Stress

The perceived psychological stress in urban built environments is often influenced by a combination of factors. Estimating and estimating a dependent variable using the optimal combination of multiple independent variables is more realistic than using a single independent variable. In short, multiple linear regression models are of greater practical relevance.

**Figure 14:**
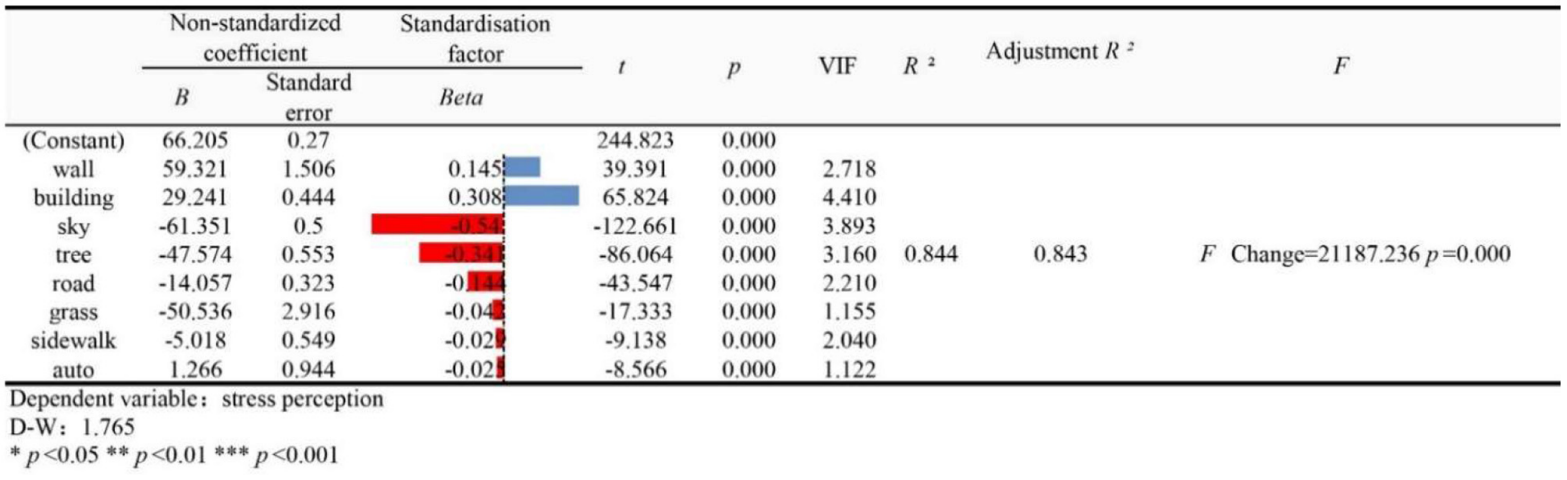
Results of linear regression analysis for perceived stress.

We employed the eight most influential visual elements in the street-view images as the explanatory variables for the perceived psychological stress of the built environment in Gangnam. The blue bars in Figure 14 represent positive correlations with perceived stress increase. The red bars depict negative correlations. The bar length represents the value of the standardized beta coefficient, and ^*^ represents the significance level. The multiple linear regression model yielded an *R*^2^ of 0.844 and an adjusted *R*^2^ of 0.843, indicating that the model explains 84.3% of the variation in the perceived psychological stress. In addition, the influence of all eight visual elements on the changes in the perceived psychological stress was significant (*p* < 0.05), while the VIFs of the model were all < 5, indicating the absence of a collinearity problem. The D–W was close to 2 (1.765), indicating the lack of autocorrelation problems and the good construction of the model. The beta coefficients for the walls and the buildings were > 0, showing that these visual elements caused urban residents to perceive psychological stress. The coefficients for the sky, vegetation, roads, grass, sidewalks, and auto were lower than 0, indicating that they lowered the psychological stress perception among urban residents.

Our research corroborated the previous researchers' findings obtained using other modalities. Using street videos and expert evaluations, a previous study concluded that the walkability of urban streets is negatively correlated with the sense of enclosure arising from the building envelope (57). The heat island effect in cities also affects the perceived stress. However, it is often difficult for urban planners to account for it because thermal comfort is generally an important factor in perceived stress (58, 59). In addition, the vegetation in urban centers in developed countries reduces the sky coverage by an average of 18.52% (60). Consequently, vegetation and sky can be considered highly correlated factors, whose influence on the perceived stress can be controlled at the urban planning level. The vegetation on the street can release oxygen and ions through respiration. These ions can regulate the function of the human cerebral cortex, such that excitation and inhibition mechanisms are balanced; fatigue is eliminated; the spirit is invigorated; and work efficiency is improved (43). This beneficial effect of vegetation on the body reduces the perception of negative emotions. Therefore, the perceived stress in urban built environments must be reduced by increasing the openness of the sky and the vegetation through regulations and micro-behavioral measures, weakening the influence of urban buildings.

## Discussion

### Measuring Urban Stress Perception Provides Scientific Help to Improve Residents' Health

Stress is a side effect of the urban construction process and is an important indicator of human health in the context of urbanization. According to research from a variety of disciplines, there is a strong correlation between perceived stress, the urban built environment, and socioeconomic conditions. However, there has been a lack of suitable data sources and scientific research methods for the quantitative analysis of the relationship between the urban built environment and citizens' perceived stress. As a possible approach to overcome this issue, GSV data currently covers a large area of most cities around the world and has become a reliable source of information for built environments. Based on this information, the perceived stress levels of urban residents can be estimated. Our study proposes a systematic approach that can effectively measure the perceived psychological stress of residents in urban built environments. This work is creating a relationship with urban planning development decision makers and mental health researchers. To collaborate research to reduce urban environmental stress. Both researchers can collect data on a large scale to measure the perception of urban stress and map the emotional distribution of stress among urban residents. Reflects perceived autocorrelation of stress in the study area. This will help urban planners to identify areas with high values of perceived autocorrelation of psychological stress. Enables researchers to provide solutions for reducing the stress of city dwellers and building more relaxing urban from the perspective of their respective disciplines and industries.

### Exploring Stress Perception Clustering Features and Visual Element Influence Mechanisms

In this study, we constructed a systematic methodological framework using GSV data and deep learning techniques to measure the state of psychological stress levels in urban spaces. Compared to traditional processes and methods, the process developed in this study is more streamlined and efficient. The systematic and innovative nature of this approach is reflected in the efficient collection of urban streetscape data through GSV and the delimitation of district boundaries, the innovative use of cloud services technology to measure the stress perception of volunteers in urban spaces, and the prediction of stress perception in urban spaces using deep learning techniques. This was followed by an empirical case study of stress perception mapping in the Gangnam district and its various wards in Seoul, Korea, to demonstrate the reliability and advantages of the proposed approach. The results show that there is a strong spatial autocorrelation in psychological stress perception in space. There are more low-low clusters in the urban traffic arteries and riverine areas of Gangnam district, and more high-high clusters in the commercial and residential areas. The analysis of the street view images for low, medium and high stress perceptions yielded the proportion of each streetscape element associated with each stress level. Finally, using multiple linear regression, we found that walls, buildings, and stress perceptions were positively correlated, while the sky, trees, and roads were negatively correlated with stress perceptions. Our work thus offers practical and innovative contributions. The correlation between the street view data and stress perceptions was validated, so we hope that the effects of changes in the visual elements of urban streetscapes on stress perceptions can be more clearly understood.

### Limitations and Future Works

Although the present study has a number of strengths, some elements should be refined in the future research. In this work, we used GSV images from Gangnam District of Seoul. However, larger scale data collection is needed to reduce the error in results. Therefore, in the future research, we will seek to gather complete streetscape data for one or more cities, which would allow cross-sectional perceived stress comparisons between cities in different countries. The increased data volume will test the predictive efficiency of the designed model. In this study, the human–machine adversarial model based on a random forest framework required human intervention for data calibration. We hope to develop deep learning tools that are based on artificial neural networks. Global urban built environment predictions can be made for the remaining data given a certain amount of training data. This is vital for large-scale multi-city perception studies.

Finally, the impact on perceived urban stress is a multifaceted process influenced by a variety of factors. Other factors influencing human's perceived urban stress should be thoroughly discussed. In the future, other factors, including the distribution of businesses and urban park point-of-interest data should be regressed against the perceived stress scores in multi-scale geographically weighted regression. This can be used to investigate pathways to reduce the perceived urban stress from a multi-source perspective.

## Conclusion

With the continuous development of society, human's psychological health is receiving more and more attention from academic community. Measuring the level of psychological stress in urban built environment based on the human perception perspective has also become a research hotspot. Due to the limitations of previous research methods, it is time-consuming and inefficient to conduct large-scale psychological stress perception surveys. With the development of computer technology, deep learning technology provides help to explore the perception of psychological stress in urban built environment. This study explores a method to describe the level of psychological stress perception in streets from a human–centered perspective by combining deep learning with spatial autocorrelation analysis. The method can provide a valid assessment of the perceived psychological stress in the built environment of cities. We believe that this innovative approach can support re-construction of the built environment on the street by facilitating the link between psychological stress perception studies and new data and technologies. It has important implications for research related to stress perception in the urban built environment.

## Author's Note

We used the latest 2021 data for the administrative divisions of South Korea available from http://www.gisdeveloper.co.kr/?p=2332.

OpenStreetMap road network data and Korean administrative data were used to obtain road network data for Gangnam District and create the GSV acquisition point coordinates. We have made these data available for download from https://www.openicpsr.org/openicpsr/project/159481/version/V1/view.

Cityscapes is an open data set that can be downloaded from the official website (https://www.cityscapes-dataset.com/).

Our research team built the SegNet neural network in Section 4 based on the Python language, Keras deep learning framework, transfer learning techniques, and TensorBoard module. The code can be downloaded from our GitHub website (https://github.com/landscapewl/Segnet-Transfer-Learning).

## Data Availability Statement

The datasets presented in this study can be found in online repositories. The names of the repository/repositories and accession number(s) can be found in the article/supplementary material.

## Author Contributions

XH and LW: conceptualization and writing—original draft. XH and SS: resources. JH and TJ: supervision. LW: validation. All authors contributed to the article and approved the submitted version.

## Funding

This work was supported by the Stable Support Programme for Higher Education by Shenzhen R&D Funds (Grant No. GXWD20201230155427003-20200803172955008).

## Conflict of Interest

The authors declare that the research was conducted in the absence of any commercial or financial relationships that could be construed as a potential conflict of interest.

## Publisher's Note

All claims expressed in this article are solely those of the authors and do not necessarily represent those of their affiliated organizations, or those of the publisher, the editors and the reviewers. Any product that may be evaluated in this article, or claim that may be made by its manufacturer, is not guaranteed or endorsed by the publisher.
